# Adaptation of an In-Person Internship to a Virtual Format for Public Health Undergraduates

**DOI:** 10.2196/35252

**Published:** 2022-03-04

**Authors:** William D Kernan, Corey H Basch

**Affiliations:** 1 Department of Public Health William Paterson University of New Jersey Wayne, NJ United States

**Keywords:** internship, remote learning, high-impact practice, COVID-19, public health education, learning outcomes, virtual learning, virtual internship, public health, health education, undergraduate education, virtual education

## Abstract

The disruption of traditional, in-person learning due to the COVID-19 pandemic necessitated the rapid development and use of revised and novel learning opportunities using a variety of remote instructional methodologies. This viewpoint describes the process used by an undergraduate Public Health program to transition a traditional, in-person, semester-long, 480-hour internship to a virtual-only learning experience guided by the existing student learning outcomes. Working closely with public health professionals at existing internship agencies, alumni from the program, student interns, and program faculty developed a modified virtual internship composed of 6 components. The development of this modified virtual internship model was guided by previous research on the components of successful internships and the elements of high-impact learning practices.

## Introduction

The COVID-19 pandemic has brought unprecedented changes for college students. As SARS-CoV-2, the virus that causes COVID-19, is primarily spread through airborne transmission, institutions of higher education were noted as communities of concern, as dormitories have close living quarters, and classrooms are often set up in a way to allow collaboration rather than social distancing [1]. This concern was increased in densely populated areas where the pandemic was more prevalent. In March 2020, colleges began sending residential students home and making a shift to web-based learning, which brought about a sense of turmoil and loss for many. As COVID-19 persists, efforts to fully open campuses across the country have been hindered. The US Centers for Disease Control and Prevention suggested ways for institutions of higher education to reopen safely, which included staggered times in classrooms, limited gatherings, and proper use of face masks, noting that the safest option was offering virtual-only learning options, remote activities, and web-based events for students and faculty [2]. Research suggests that these changes evoke a series of negative outcomes such as increased stress, anxiety, loneliness, academic burden [3,4]. In addition, closures and format pivots have led to lost opportunities for students, such as jobs and internships, as well as countless other challenges [5].

In response to the issues that arise due to what has been lost during the pandemic, many efforts have been made to retain high-quality and engaging learning experiences in a virtual format. In addition to coursework, other examples include experiences such internships, which can better equip students for the workforce upon graduation. As many students have yet to gain this experience, the value of maintaining internship opportunities cannot be understated. More than half of graduating college students in the United States participated in at least 1 internship during their academic training [6]. Internship experiences are important, as research indicates that they can promote strong university-community partnerships [7]; they can improve interns’ knowledge, research skills, and communication skills; and they can result in products that are useful to the internship site [8].

### Purpose

The purpose of this viewpoint is to describe the process and experience of transitioning a traditional (in-person) internship to a virtual experience in the Public Health undergraduate program at a public university in New Jersey. The traditional internship is a 12-credit course where interns complete (1) an in-person, classroom-based, didactic experience; and (2) an in-person field placement.

### Components of the Traditional Internship

#### Didactic Component

Prior to the semester in which the field placement occurs, students complete a 1-credit Introduction to Internship course. This in-person seminar-style course is designed to assist students in the development of the professional skills necessary for the successful completion of an internship in public health education. Topics of study include professional conduct, professional communication, resume development, interviewing skills, and job search strategies. Concurrent to the course, students work closely with the internship site coordinator to review the various types of public health education field placement settings and select their placement for the following semester. The internship site coordinator is a professional staff member who works in the academic department to coordinate and supervise the field placements.

During the 12-credit field placement semester, interns participate in classroom-based instruction necessary for the completion of a Capstone Project and faculty-led review for the certified health education specialist examination, administered by the National Commission for Health Education Credentialing, which interns take at the end of the semester. The Capstone Project provides interns the opportunity to demonstrate their understanding of public health education program planning and is the final assessment of their ability to develop a comprehensive health education program from conception through assessment, planning, implementation, and evaluation. While preparation for the certification exam does not demonstrate an intern’s potential professional competence, it does provide both the faculty member and the intern an important opportunity to gauge the intern’s application and interpretation of knowledge.

#### Field Placement

The field placement is a supervised experience designed to allow interns to apply their newly acquired knowledge and skills in a professional work setting. The field placement occurs at an approved public health agency in the community where the intern works under the supervision of a public health professional, the internship site supervisor. Student learning outcomes center on the five following major themes: (1) exposure to the roles and responsibilities of an entry level public health educator in a public health agency; (2) examination of the ways in which theoretical concepts are applied to the realities of the field of public health education; (3) exploration of strategies for communicating and working with public health professionals; (4) self-reflection regarding career goals and lifelong learning; and (5) improvement of public speaking and audience management skills.

Interns spend approximately 28-30 hours per week at their field placement over the course of 15 weeks. During this time, interns work with their internship site supervisor, other partners at the agency, and stakeholders in the community to complete activities and projects that fall within the professional scope of practice for entry-level health education specialists. During this experience, 2 site visits are conducted by the internship site coordinator to review the intern’s progress toward meeting the student learning outcomes of the field placement portion of the internship experience.

As the pandemic changed the nature of in-person instruction for institutions of higher education, an alternate structure for the internship needed to be developed. It was necessary that the new structure include robust activities and rich experiences that would substantively address the 5 student learning outcomes. The following is a description of the 6 components that were included in the virtual internship experience in the spring semester of 2021 to accommodate all students and provide what proved to be an experiential learning opportunity. A comparison of the components of traditional and virtual internships is displayed in Table 1.

**Table 1:** Components of traditional and virtual internships.

Internships and components		Activities completed by intern
**Traditional internship**		
	Didactic component (in-person)	Capstone Project Directed faculty-led preparation for credentialing exam
	Field placement	In-person placement project
**Virtual internship**		
	Didactic component (web-based)	Capstone Project Directed faculty-led preparation for credentialing exam
	Field placement	Remote placement Internship Site Project (new)
	Professional development points (new)	Minimum of 40 hours of professional development
	Alumni mentorship program (new)	Minimum of 8 mandatory mentorship sessions

### Components of the Virtual Internship

#### Revised Didactic Component

The previously described faculty-led coursework was moved to a fully web-based format and maintained focus on the completion of the Capstone Project and preparation for the certification exam. Instruction for completing the Capstone Project was modified to suit the web-based learning environment. It included weekly content review using the University’s web-based learning management platform, several required individual feedback sessions with the faculty member, and the frequent review of each intern’s progress toward completion of the project. Web-based exam preparation consisted of faculty-supervised web-based study groups that met several times each week, weekly virtual practice exams, and faculty-recorded review sessions for each weekly exam.

#### Remote Field Placement

The Introduction to Internship course completed by students the semester prior to their field placement was moved to a web-based format and retained all the course content previously described. The main challenge during this semester was the identification of public health agencies that were willing and able to work with interns virtually. The internship site coordinator queried all existing internship site supervisors to determine if their agency had the ability to host an intern during the pandemic. As remote field placement sites were identified, it became apparent that many field placement sites would not have the capacity for a semester-long field placement, as was the case in prepandemic years. Therefore, the faculty decided to proceed with a shortened field placement experience that would be completed in 1 month, rather than the full semester. Furthermore, to increase the likelihood of meeting student learning outcomes and better manage the remote placements, fewer field placement sites were selected. Those that were selected agreed to take a different intern each month (1 each in February, March, and April). As a result, instead of having to recruit 60 unique field placements, only 20 were needed. This structure provided multiple benefits as the internship site supervisors were able to develop a 4-week pattern of learning activities for the intern and then replicate that same pattern during the subsequent 2 months. Furthermore, this allowed the faculty to hand select those public health agencies who were best positioned to provide robust learning experiences in a truncated format. Finally, the new structure of the field placement assisted the internship site coordinator to quickly identify and rectify any emergent issues during an intern’s field placement. This last point is particularly important because issues needed to be addressed quickly as the duration of the field placement was only 4 weeks.

Once field placement sites were identified, interns were assigned to a public health agency that aligned with their interests. Similar to what occurs during a traditional internship, during this virtual site rotation, interns met several times each week with agency staff and their internship site supervisor, completed assigned tasks, developed public health projects, learned about how the agency functions, attended work-related meetings, and engaged in other related learning activities. During the field placement, interns also worked on an Internship Site Project, a new addition to the virtual internship created to ensure that interns would have the opportunity to demonstrate the community-based health program planning principles that are central to the program’s learning outcomes. The topic of this project was identified during the first 2 days of the field placement in consultation with the intern’s site supervisor. The project was a collaboration between the intern and the agency and could be on any of the following topics: “Press release,” “Strengths/ Weaknesses/Opportunities/Threats analysis,” “PowerPoint presentation,” “Brochure,” “Infographic,” “Data analysis summary report,” “Logic model,” “Flyer,” “Website development,” and “Social-media posting.”

#### Professional Development Points

Due to the shortened and web-based nature of the field placement, the faculty were concerned about the loss of opportunities for professional development, as the potential to attend professional meetings and conferences was limited during the pandemic. To highlight the need for public health educators to remain updated on current public health information and developments and to emphasize the importance of lifelong learning, all interns engaged in professional development activities in the form of online trainings, webinars, and live virtual conferences. Interns earned “Professional Development Points” by participating in professional development opportunities selected from a faculty-created course catalog of free online opportunities offered by regional public health training centers, state and local health departments, and federal health agencies, such as the Centers for Disease Control and Prevention’s Train network.

When selecting professional development opportunities to include in the catalog, the faculty focused on choosing opportunities that reinforced issues around diversity, health equity, and social justice, as these topics are central to the program’s learning outcomes. Furthermore, opportunities for learning about topics that are not fully covered in the undergraduate curriculum (eg, maternal and child health, emergency preparedness, bioterrorism, and occupational health) were also included in the training catalog. The interns were required to complete a minimum of 40 hours of training, with 1 hour of training equivalent to 1 professional development point. As evidence of the completion of a professional development opportunity, the interns were required to complete a post-training evaluation to earn a Certification of Completion, which was then uploaded to their e-portfolio.

#### Alumni Mentorship Program

While the interns would be in contact with their internship site supervisor several times each week during their 4-week field placement, the faculty were concerned about the lack of periodic mentorship during the 2 months when the intern was not assigned to a field placement. To provide a consistent point of contact during the whole of the semester, each intern was assigned to a successful alumnus who served as their mentor. Alumni mentors were recruited by the program faculty the semester prior to field placement. All alumni mentors participated in an online training prior to the start of the field placements.

Over a 12-week period during the spring semester, the intern and the mentor met at least 8 times to review a predetermined set of discussion topics designed to help the intern gain a deeper understanding of what it is like to work in the public health field. Meetings with mentors were designed to help the intern better understand the transition from college to the professional public health work environment and identity the major issues that arise when working in the public health field. To facilitate these meetings, 8 discussion guides, which acted as a set of guiding questions that the intern should ask their mentor, were created. There was 1 discussion guide for each of the 8 required meetings. At the end of the semester, both the intern and the mentor provided written evaluations of this experience.

#### e-Portfolio

To provide a directed opportunity for self-reflection, career exploration, and preparation for the job search, all interns were required to compile an e-portfolio using the free web-based software system, WIX (Wix.com Inc). Training on how to develop the e-portfolio was provided by a representative from WIX, and supervision of this component of the internship was provided by the internship site coordinator.

An e-portfolio serves several purposes. First, it is a digital compilation of student work upon which they can reflect on the tangible work they have produced. Second, it serves as a valuable means of assessment for faculty to comment on the strengths of the student’s work and areas for improvement. e-Portfolios can be accessed globally and are highly visible, a limitation of traditional portfolios. As they are easily accessible, e-portfolios can be shared with mentors, colleagues, and family members for review and comment [9]. Finally, e-portfolios can serve as a tool for students to compete for jobs after graduation. In their e-portfolio, the interns were required to include a resume, examples of prior classwork from preselected public health courses, the Internship Site Project, all earned professional development point certificates as proof of meeting the minimum number of required points, and the Capstone Project.

## Discussion

### Structuring the Virtual Internship

As the components of the virtual internship were being developed, careful attention was given to identifying what learning opportunities might be compromised in a virtual-only format and how program student learning outcomes would be met with revised and new activities. The shift of the fieldwork experience to the web-based environment called into question the quantity and quality of the interpersonal aspects that a traditional, in-person internship offers to student interns. The areas of primary concern were potentially lost opportunities to network, to be mentored by a professional within a physical work environment, and to demonstrate the skills and competencies necessary for entry-level health education practice.

The model that was developed for the remote internship experience considered these factors. The faculty wanted to offer experiences that, as best as possible, would mimic traditional work settings while at the same time offering alternate experiences for networking and mentoring; it also aimed to enable the intern to reflect on their skills and competencies and prepare for job search. As the importance of professional development and lifelong learning may be lost in the virtual context, it was critical that a professional development component be created for the virtual internship experience.

While developing the structure of the virtual internship, 2 frameworks were used to guide the selection of activities and experiences to ensure that the virtual internship would offer a high-quality experience that allowed interns to achieve the intended learning outcomes.

### A “Pedagogy of Internships”

King and Sweitzer [10] describe 4 dimensions of learning and development that comprise a “pedagogy of internships.” These include professional, academic, personal, and civic dimensions. While each of these dimensions is readily apparent within and intentionally built into the traditional, in-person internship, ensuring that each dimension was adequately addressed was a priority during the revision process.

The professional dimension of an internship allows for the exploration of career pathways, the application of skills in professional settings, and the opportunity to observe the patterns of work and behavior that occur in a potential work setting [10]. In the virtual internship model, this dimension is mainly addressed by the field placement experience and alumni mentorship program. The remote field placement exposes interns to the professional dimension directly through activities conducted by the agency to which the intern is assigned. Examples of these activities include virtual opportunities to interact with coworkers, attending online meetings, participating in the agency’s programs, and receiving supervision from the internship site supervisor. Furthermore, the mentorship provided by the alumnus who works in the profession provides the opportunity to process the field placement experiences and explore career opportunities through a series of directed mentorship sessions.

The academic dimension of the internship focuses on the development of patterns of thinking that are consistent with the academic discipline, resulting in the intern’s ability to think, in this case, like a health education specialist [10]. The academic dimension of the virtual internship is addressed by several components of the internship but is most clearly evidenced through the work that interns complete during the didactic component of the internship. Working closely with a faculty member, interns identify a topic for their Capstone Project and fully develop a public health education intervention from conception to completion. At the same time, the interns are involved in concentrated certification exam preparation, which serves to reinforce the concepts, frameworks, and skills necessary to think like a health education specialist. The interns are introduced to the importance of continuing education and lifelong learning through the professional development point requirement. Finally, the completion of the Internship Site Project during the field placement is yet another activity where the academic dimension of the internship is embedded.

The third dimension of an internship, the personal dimension, centers on the exploration of life skills beyond those necessary for professional practice. This includes the development of intellectual and emotional qualities such as flexibility, openness to differences, self-awareness, self-efficacy, and sound judgement [10]. The personal dimension is first addressed in the Introduction to Internship course. This experience exposes the student to many of the concepts related to working with others and effective communication. The virtual internship, through the field placement and mentorship components, supports interns in the continued exploration of these concepts. Topics of the mandatory mentorship sessions, for example, include goal setting, building rapport with coworkers, taking initiative, exploring conflict, and dealing with professional challenges. While the development of the e-portfolio provides interns an opportunity to document their academic achievements, it also allows interns to communicate other facets of their identity, such as hobbies, interests, personality characteristics, and life experiences.

The civic dimension addresses the ways in which professionals work with and serve society through their professional role [10]. This dimension was perhaps the area for which the faculty had the greatest concern when pivoting to the virtual format. The loss of three-quarters of the in-person time at the field placement called into question the quantity and quality of the interactions that interns would have with the individuals and populations served by the agency. As community engagement is a cornerstone of public health education practice, developing a mechanism for interns to engage in intentional ways with their constituents was critical. In the virtual internship, this was addressed through the completion of the Capstone Project and with the new requirement for the development of the Internship Site Project. The Capstone Project requires students to conduct a community needs assessment using multiple methods. Students engage in civic involvement through this process as they organize a program planning committee consisting of community members and involve the priority population in key informant interviews and focus groups. Observational research provides interns the opportunity to traverse the community as they complete windshield and walking surveys. As the pandemic limited the exposure to the community that interns typically have when completing these activities, the new Internship Site Project required that the intern work closely with their internship site supervisor to identify a discrete project that would directly involve the community in some way, while meeting some goal of the agency where the intern was placed. Examples of remote community involvement activities in which interns engaged include reviewing community needs assessment data with key stakeholders, involving community members in online planning meetings, creating digital health education materials with community input, health messaging via the field placement site’s social media platforms, moderating web-based conferences, presenting data at virtual community coalition meetings, and working with community members to develop program evaluation instruments.

While moving toward the completion of the virtual internship model based on these 4 dimensions of learning and development, the faculty felt it was important to ensure that the new internship model remained a high-impact practice. Internships are routinely identified as being a high-impact practice. A high-impact educational practice is any teaching or learning experience that education research has shown to have a positive impact on students [11]. This analysis is relevant to those training health educators as well as public health agencies who will be hiring and working with these future professionals. Understanding what an intern may or may not have experienced during an internship can be helpful in determining future professional development needs.

### Virtual Internship as a High-Impact Practice

A high-impact practice features 6 common elements. First, it requires work, where students are expected to engage in activities that require considerable time and effort. Next, it requires that students engage with others is an intentional way, including faculty, peers, supervisors, and coworkers. A high-impact practice also requires that students interact with and develop ways of working with individuals who are different from themselves. The provision of quality and frequent feedback on performance in both formal and informal manners is another element of a high-impact practice. Students must also be provided with opportunities for the application of knowledge in new situations. Finally, a high-impact practice must provide students with the opportunity for self-reflection on their personal and professional development [12].

While each component of the virtual internship addresses most of the elements of a high-impact practice to some degree, it was important for the faculty to identify where they are addressed in a substantive and intentional manner. Table 2 provides a visual display of the components of the virtual internship that substantively addresses the 6 elements of a high-impact practice.

**Table 2:** Virtual internship as a high-impact practice.

	Capstone Project	CHES^a^ exam preparation	Remote field placement	Professional development	Alumni mentorship	e-Portfolio
HIP^b^s are effortful	✓	✓	✓	✓	✓	✓
HIPs help students build substantive relationships	✓		✓		✓	
HIPs help students engage across differences	✓		✓	✓	✓	
HIPs provide students with rich feedback	✓	✓	✓		✓	
HIPs help students apply and test what they are learning in new situations	✓		✓			
HIPs provide opportunities for students to reflect on the people they are becoming			✓	✓	✓	✓

^a^CHES: certified health education specialist.

^b^HIP: high-impact practice.

### Limitations

While conversion of a traditional internship to a virtual format described above maintained the key elements of a high-impact educational practice, there are some limitations to an exclusively online internship that deserve attention. First, revision of the traditional internship program into a virtual format was challenging as most elements of a virtual internship naturally rely on the use of remote technologies. Varied technologies were used in this virtual internship model, including campus-based learning management platforms, webinars, various presentation software, the opportunity to attend meetings and conferences online, working on team projects virtually, and meeting with mentors via social media platforms. Selection of specific technologies must consider issues such as accessibility, internet speed, as well as hardware and memory capacity. Alternate arrangements for students who do not have the necessary level of accessibility must be made available.

Furthermore, in a virtual internship experience, interns spend most of their time in front of a computer screen engaging in the various components of the program. As a result, the types of interpersonal interactions that naturally occur in an in-person environment are different in a virtual setting. This presents a particular challenge to educators when attempting to identify learning activities that closely replicate the work environment. Further research in this area is needed.

Moreover, as the intern is not able to engage with the community served by the field placement site in traditional ways, it is uncertain if the intern is fully able to understand the needs of the community. Therefore, a virtual internship must intentionally include opportunities for interaction with faculty, peers, mentors, and colleagues, as well as opportunities for deep engagement with community members so that interns develop a greater understanding and empathy for the individuals in the community served by the field placement site.

Finally, as remote field placement sites were identified, it became apparent that a semester-long experience would not be possible, as most internship site supervisors indicated that they would not have the capacity for such a time-intensive field placement. This loss of contact hours is a limitation for which alternate activities needed to be identified. Mentorship opportunities and the emphasis on engagement in professional development opportunities were 2 ways in which the loss of contact hours was addressed in the virtual internship described in this viewpoint. The development of additional activities to address this loss of contact hours is warranted.

### Suggestions for Further Study

There is no doubt that technology in educational settings will continue to play a major role in student learning. In recent decades, there has been accumulating evidence supporting the value of interventions delivered through digital technology, including but not limited to texting, telecommunications, and real time monitoring of symptoms and emotions [13-16]. Consequently, there is a need for individuals to learn and apply such technologies to help people make informed decisions about individual and community health [17-20]. The virtual internship, while prompted by necessity due to the COVID-19 pandemic, resulted in a wide range of benefits for students.

The benefits of a virtual internship include the ability for students to learn and practice skills related to communication with technology, the availability for interaction with supervisors with convenient timing and modalities, more frequent contacts at lower costs, and adapting to different (and potentially challenging) implementation contexts [21,22]. These benefits proved to be true in the development of the virtual internship described in this viewpoint.

In addition, the virtual internship experience enabled students to learn and practice new skills and for community members to benefit from education and outreach while helping to ensure the safety of students and the community, as well as reducing travel costs and increasing convenience. Finally, while the need for virtual learning opportunities were prompted by the COVID-19 pandemic, it seems almost certain that this need will likely persist, which increases availability and access to public health education for some populations who would otherwise suffer consequences from unanswered questions, and for students to become adept at skills that will be needed going forward [23].

### Conclusion

The existing traditional undergraduate public health internship described in this viewpoint has undergone many modifications in its nearly 40-year history. However, no past revision was as extensive, necessary, or rapid as the transition to a virtual internship due to the COVID-19 pandemic. Guided by conceptual frameworks and the sincere desire on the part of the faculty to ensure that students received a robust learning experience, a virtual internship experience was developed that provided a well-organized and high-impact learning opportunity.
